# Autoantibodies to central nervous system neuronal surface antigens: psychiatric symptoms and psychopharmacological implications

**DOI:** 10.1007/s00213-015-4156-y

**Published:** 2015-12-14

**Authors:** T. A. Pollak, K. Beck, S. R. Irani, O. D. Howes, A. S. David, P. K. McGuire

**Affiliations:** Department of Psychosis Studies, Institute of Psychiatry, Psychology and Neuroscience, King’s Health Partners, King’s College London, De Crespigny Park, Denmark Hill, London, SE5 8AF UK; Nuffield Department of Clinical Neurosciences, John Radcliffe Hospital, Oxford, UK

**Keywords:** Antibody, Immunoreactivity, Inflammation, Receptor, NMDA receptor, Potassium channel, GABA receptor, Limbic system

## Abstract

**Rationale:**

Autoantibodies to central nervous system (CNS) neuronal surface antigens have been described in association with autoimmune encephalopathies which prominently feature psychiatric symptoms in addition to neurological symptoms. The potential role of these autoantibodies in primary psychiatric diseases such as schizophrenia or bipolar affective disorder is of increasing interest.

**Objectives:**

We aimed to review the nature of psychiatric symptoms associated with neuronal surface autoantibodies, in the context of autoimmune encephalopathies as well as primary psychiatric disorders, and to review the mechanisms of action of these autoantibodies from a psychopharmacological perspective.

**Results:**

The functional effects of the autoantibodies on their target antigens are described; their clinical expression is at least in part mediated by their effects on neuronal receptor function, primarily at the synapse, usually resulting in receptor hypofunction. The psychiatric effects of the antibodies are related to known functions of the receptor target or its complexed proteins, with reference to supportive genetic and pharmacological evidence where relevant. Evidence for a causal role of these autoantibodies in primary psychiatric disease is increasing but remains controversial; relevant methodological controversies are outlined. Non-receptor-based mechanisms of autoantibody action, including neuroinflammatory mechanisms, and therapeutic implications are discussed.

**Conclusions:**

An analysis of the autoantibodies from a psychopharmacological perspective, as endogenous, bioactive, highly specific, receptor-targeting molecules, provides a valuable opportunity to understand the neurobiological basis of associated psychiatric symptoms. Potentially, new treatment strategies will emerge from the improving understanding of antibody-antigen interaction within the CNS.

## Introduction

Over the last decade, there has been an increasing recognition of central nervous system (CNS) syndromes associated with autoantibodies to CNS cell surface antigens (‘neuronal surface autoantibodies’ or ‘NSAbs’). The majority of these syndromes feature prominent psychiatric and cognitive symptoms, amongst manifold neurological manifestations such as seizures, movement disorders and autonomic dysfunction and are best described as ‘autoimmune encephalopathies’. Recognition of these syndromes and research on the mechanisms of action of their associated, and likely pathogenic, antibodies has had a huge impact on clinical neurology and an increasing influence on psychiatry as well.

Given the almost universal occurrence of psychiatric and cognitive symptoms in these autoimmune encephalopathies, NSAbs are of considerable interest to researchers studying the neurobiological bases of psychiatric symptoms. In recent years, the possibility that NSAbs can cause a ‘purely psychiatric’ phenotype has been the object of interest, partly because of the implication that were this to be the case at least a subset of what is currently termed primary psychiatric disease may in fact be NSAb-mediated and potentially respond to treatment with immunotherapies (Deakin et al. 2014) (Table 1).

**Table 1:** Neurological and psychiatric associations of NSAbs

Antigen	Antigen description/epitope	Main encephalopathy syndrome; which psychiatric features?	Other associated neurological disorders	Antibodies in isolated psychiatric syndromes
NMDAR	Ligand-gated ion channel ATD of NR1 subunit (Gleichman et al. 2012) (note NR2 Abs identified in SLE (Lauvsnes and Omdal 2012))	Encephalopathy (usually extralimbic) Psychiatric features include anxiety, agitation, bizarre behaviour, catatonia, delusional or paranoid thoughts and visual or auditory hallucinations. Also movement disorder, seizures and autonomic instability (Kayser et al. 2013; Irani et al. 2010a; Titulaer et al. 2013)	Post-herpes simplex encephalitis relapse with chorea; idiopathic epilepsy; immunotherapy-responsive dementia (Pruss et al. 2012a; Doss et al. 2014)	Case reports and series including psychosis, autism, BPAD and eating disorders (Choe et al. 2013; Zandi et al. 2014; Heresco-Levy et al. 2015; Creten et al. 2011; Mechelhoff et al. 2015; Perogamvros et al. 2012) Prevalence: Meta-analyses: schizophrenia 7 % (IgG, A + M), only FEP greater than controls (Pollak et al. 2014); odds ratio of seropositivity in schizophrenia, schizoaffective disorder, BPAD or MDD vs controls = 3.1 (Pearlman and Najjar 2014) but note subsequent studies showing zero prevalence (Masopust et al. 2015; de Witte et al. 2015) or no difference from controls (Dahm et al. 2014; Steiner et al. 2014) Treatment-refractory psychosis 7 % (Beck et al. 2015); post-partum psychosis 2 % (Bergink et al. 2015); paediatric psychosis 14 % (Pathmanandavel et al. 2015); borderline personality disorder 2.4 % (Dahm et al. 2014)
LGI1	VGKC- and AMPAR-associated secreted molecule	LE with or without faciobrachial dystonic seizures Psychiatric features include confusion, hallucinations and depression (Irani et al. 2010b)	Morvan’s syndrome, NMT, epilepsy, REM sleep behaviour disorder (Irani et al. 2010b)	Prevalence: Schizophrenia 0.1 %; not found in affective disorders or borderline PD (Dahm et al. 2014)
CASPR2	VGKC-associated adhesion molecule Multiple epitopes in extracellular domain including one in discoidin-like domain and in laminin module (Olsen et al. 2015; Pinatel et al. 2015)	Morvan’s syndrome Psychiatric features include confusion, hallucinations, agitation and delusions (Irani et al. 2011)	LE, NMT, epilepsy (Irani et al. 2010b)	Prevalence: Schizophrenia 1.5 %; affective disorder 0.6 %, not found in borderline PD (Dahm et al. 2014)
AMPAR	Ligand-gated ion channel Bottom lobe of ATD (Gleichman et al. 2014)	LE Psychiatric features include confusion, personality change, psychosis, apathy, agitation and confabulation (Lai et al. 2009; Hoftberger et al. 2015; Dogan Onugoren et al. 2014)	n/a	Case reports (Graus et al. 2010; Elamin et al. 2015) Not found in schizophrenia, affective disorders or borderline PD (Dahm et al. 2014)
GABA_A_R	Ligand-gated ion channel α_1_ or β_3_ subunits (Petit-Pedrol et al. 2014) α_1_ and γ_2_ subunits (Pettingill et al. 2015)	LE with refractory seizures Psychiatric features include confusion, affective changes (inc depression) and hallucinations (Petit-Pedrol et al. 2014)	Varied presentations (Pettingill et al. 2015)	Cases described with predominant anxiety and catatonia (Pettingill et al. 2015)
GABA_B_R	Ligand-gated ion channel	LE with refractory status epilepticus Psychiatric features include psychosis, agitation and catatonia (Dogan Onugoren et al. 2014; Lancaster et al. 2010)	Opsoclonus-myoclonus; cerebellar ataxia; PERM (Lancaster et al. 2010; Hoftberger et al. 2013)	Prevalence: 0.3 % of affective disorder (Dahm et al. 2014)
D2R	Metabotropic receptor IgG from SC but not PANDAS binds to extracellular epitope at ATD (Cox et al. 2013)	‘Basal ganglia encephalitis’ with prominent movement disorder (dystonia, parkinsonism, chorea, tics) Psychiatric features include agitation, depression, psychosis, emotional lability.	SC, PANDAS (Cox et al. 2013)	Prevalence: Paediatric psychosis 7 % (Pathmanandavel et al. 2015); Tourette syndrome 9 % ((Dale et al. 2012); not found in acute exacerbation of established schizophrenia (Muller et al. 2014)
DPPX	Auxiliary subunit of Kv4.2 potassium channels	LE with enteropathy Psychiatric features include amnesia, delirium, psychosis and depression (Boronat et al. 2013; Tobin et al. 2014)	PERM (Balint et al. 2014)	Prevalence: 0.1 % of schizophrenia (Dahm et al. 2014)
MGluR5	Metabotropic receptor	‘Ophelia syndrome’: LE in association with Hodgkin lymphoma. One case of LE without lymphoma. Psychiatric features include depression, anxiety, delusions, visual and auditory hallucinations, anterograde amnesia (Lancaster et al. 2011)	n/a	Prevalence: 0.1 % of schizophrenia (Doss et al. 2014)

*ATD* amino terminal domain, *BPAD* bipolar affective disorder, *CBA* cell-based assay, *ELISA* enzyme-linked immunosorbent assay, *LE* limbic encephalitis, *MDD* major depressive disorder, *NMT* neuromyotonia, *PERM* progressive encephalomyelitis with rigidity and myoclonus, *RIA* radioimmunoassay, *SC* Sydenham’s chorea, *PANDAS* paediatric autoimmune neuropsychiatric disorders associated with streptococcal infections, *n/a* not applicable

### Technical developments

The methodology that has facilitated the last decade’s rapid increase in research has been the development of cell-based assays (CBAs) using human embryonic kidney (HEK) cells that have been transfected to express the antigen of interest on their surface (Rodriguez Cruz et al. 2015). These assays have a number of advantages over the immunoassays that preceded CBAs, such as enzyme-linked immunosorbent assay (ELISA) or radioimmunoassay (RIA). Firstly, the antigenic target is presented in its native conformation at the cell surface: antibodies which target such proteins are likely to operate in vivo. Secondly, antibodies that can be demonstrated to target extracellular antigens are likely to be pathogenic (Graus and Dalmau 2012). Not surprisingly, therefore, the disorders associated with NSAbs detectable via CBAs, unlike those associated with the classical intracellularly directed onconeural antibodies, tend to be immunotherapy-responsive, with sometimes even the most acutely unwell patients making a substantial or even complete recovery following immunotherapy (Kayser et al. 2013).

### The patient’s autoantibodies as pseudo-pharmacological agents: a new paradigm in psychopharmacology

NSAbs form a unique class in that their clinical expression is at least in part mediated by their effects on neuronal receptor function, primarily at the synapse; usually, this results in receptor hypofunction (see Table 2). Although these effects are not thought to occur primarily via direct action of the antibody at the receptor, as is the case with psychotropic drugs, this general mechanism of action invites an analysis of the antibodies from a pharmacological perspective, as endogenous, bioactive, highly specific, receptor-targeting molecules. This concept builds on the established notion of an ‘autoimmune channelopathy’; but while some NSAbs do target ion channels (e.g. *N*-methyl-d-aspartate receptor [NMDAR], α-amino-3-hydroxy-5-methyl-4-isoxazolepropionic acid receptor [AMPAR], γ-aminobutyric acid receptor [GABAR]), it should be noted that others are specific for receptor-associated or regulatory molecules (e.g. contactin-associated protein-like 2 [CASPR2], leucine-rich glioma inactivated 1 [LGI1], dipeptidyl-peptidase-like protein-6 [DPPX]) or metabotropic cell surface receptors (e.g. dopamine D2 receptor [D2R], metabotropic glutamate receptor 5 [mGluR5]). Mutations in many of these proteins have been linked to neuropsychiatric conditions, strengthening the likelihood of antibody pathogenicity (Irani et al. 2014).

**Table 2:** Functional effects of NSAbs

Antigen	Anatomical binding specificity	Direct receptor/antigen action?	Effects on receptor/antigen distribution	Effects on currents and synaptic plasticity	Complement deposition/inflammation	Other effects	Non-IgG subtypes
NMDAR	Hippocampus > cortex, striatum + cerebellum (Moscato et al. 2014)	None (Moscato et al. 2014)	Internalised; reversible reduction in cluster density (Dalmau et al. 2008; Hughes et al. 2010); surface receptors laterally displaced out of synapse (Mikasova et al. 2012); trafficked through recycling endosomes and lysosomes (Moscato et al. 2014); similar NMDAR reduction on excitatory and inhibitory neurons (Moscato et al. 2014)	Decreased synaptic NMDAR-mediated currents (Hughes et al. 2010); LTP blocked (Zhang et al. 2012); glutamatergic synaptic plasticity blocked (Mikasova et al. 2012)	Abs bind complement in vitro but not found in post-mortem brains (Martinez-Hernandez et al. 2011)	Increase in extracellular glutamate (Manto et al. 2010); NMDAR subunit gene expression changes not seen (Moscato et al. 2014); reduction in strength of interaction between NMDAR and ephrin-B2 receptors (Mikasova et al. 2012)	IgA reduces density of NMDAR, synaptophysin, synapsin and glutamate transporters VGLUT1 and VGAT; IgA reduces NMDAR-mediated currents (Pruss et al. 2012a); IgM reduces cell survival and NR1 expression (Choe et al. 2013)
AMPAR	Hippocampus, subiculum, caudate-striatum, and molecular layer of cerebellum > other cerebrum, cerebellum and brainstem (Lai et al. 2009)	n/a	Internalisation and degradation; reduction in total surface amount and synaptic localization of GLuR1 and GLuR2 containing AMPARs (Lai et al. 2009; Peng et al. 2015) Internalised Ab complexes are localised to early endosomes, recycling endosomes and lysosomes (Peng et al. 2015)	Decreased AMPAR-mediated currents (Gleichman et al. 2014; Peng et al. 2015)	n/a	Homeostatic decrease in inhibitory synaptic strength; significant changes in the patterns of action potential firing in neurons (Peng et al. 2015)	n/a
LGI1; CASPR2 (VGKC-‘complexed’ proteins)	LGI1: neuronal cell bodies throughout the CNS including thalamic neurons and the orexin neurons of the hypothalamus, also neurons in the LC and the raphe nucleus. CASPR2: mainly in the neuropil and juxtaparanodes throughout CNS, including the thalamus, hypothalamus, LC and raphe (Irani et al. 2012)	n/a	LGI1: Abs from LE block binding of LGI1 to ADAM22—effect not seen with Abs from NMT; reversible reduction in synaptic AMPAR density (Ohkawa et al. 2013)	LGI1: Increased spontaneous depolarisations in hippocampal CA3; enhanced hippocampal mossy fibre to CA3 pyramidal cell transmission (Lalic et al. 2011)	VGKC: Post-mortem evidence of complement-mediated cell death (Bien et al. 2012) LGI1: Complement deposition in post-mortem brains (Bien et al. 2012; Klang et al. 2014)	CASPR2: IgG4 Abs from LE reduce hippocampal synaptic gephyrin clusters/disrupt inhibitory synaptic contacts of GABAergic neurons (Pinatel et al. 2015)	n/a
Dopamine D2R (note some studies have used anti-streptococcal antibodies that *cross-react* with D2R but also with other antigens)	Striatum, thalamus, frontal cortex (Brimberg et al. 2012)	Possible: IgG from SC and PANDAS subjects binds to D2R and induces inhibitory signalling comparable to dopamine (Cox et al. 2013)	n/a	n/a	n/a	In vivo effects of GAS immunisation on mouse behaviour are blocked by haloperidol and paroxetine (Brimberg et al. 2012); GAS exposure increased dopamine in medial frontal cortex and basal ganglia and decreased glutamate levels in medial frontal cortex (Brimberg et al. 2012)	n/a
DPPX	Cerebellar granular layer; hippocampal mossy fibres; cortex; striatum; ganglionic neurons in myenteric plexus of gut wall (Tobin et al. 2014)	Rapid onset of changes to neuronal firing suggests possible direct action (Piepgras et al. 2015)	Reduction of cell surface DPPX and Kv4.2 VGKCs in hippocampal neurons (Piepgras et al. 2015)	Increased guinea pig myenteric plexus/human submucus plexus neuronal firing; more neurons firing and higher frequency firing (Piepgras et al. 2015)	n/a	n/a	n/a

GABA_A_R Abs specific for α_1_ or β_3_ subunits cause reduction in synaptic surface expression of GABA_B_R with no effect on NMDAR or gephyrin (Petit-Pedrol et al. 2014). Abs specific for α_1_ and γ_2_ subunits cause reduction in surface expression of GABA_A_R (Pettingill et al. 2015). GABA_B_R: Abs bind to thalamus > hippocampus, cortex and striatum (Jeffery et al. 2013)

*GAS* group A streptococcus, *LC* locus coeruleus, *LE* limbic encephalitis, *NMT* neuromyotonia, *LTP* long-term potentiation, *VGKC* voltage-gated potassium channel, *n/a* not assessed

This review will focus on those CNS-directed NSAbs that have been associated with clinical syndromes which feature prominent psychiatric features. Some NSAbs have been more consistently associated with a particular disease phenotype than others, although with the passage of time, the number of conditions in which all NSAbs have been identified continues to increase (Irani et al. 2014).

The neurological signs and symptoms associated with NSAbs have been given less focus in this article to make space for discussion of psychiatric phenomena; we would direct readers to the article by Varley and colleagues (Varley et al. 2015) for a more neurologically focussed review.

## *N*-methyl-d-aspartate (NMDA) receptor antibodies

IgG antibodies to the extracellular N-terminal domain of the NR1 subunit of the NMDAR are associated with NMDAR antibody encephalitis. First described in 2007 in young women presenting with neuropsychiatric symptoms in the presence of an ovarian teratoma (Dalmau et al. 2007), NMDAR encephalitis has a characteristic progression, frequently involving a viral prodrome followed by two phases of illness. The early phase includes psychiatric symptoms, cognitive dysfunction and seizures, progressing later to movement disorder, dysautonomia and coma (Irani et al. 2010a). NMDAR antibody encephalitis is increasingly described in older patients, patients without tumours, men and children (Titulaer et al. 2013).

### The nature of psychiatric symptoms associated with NMDAR NR1-directed antibodies

NMDAR antibodies are of particular interest in psychiatric research, as approximately 80 % of adults with NMDAR antibody encephalitis initially present with behavioural and psychiatric symptoms (Kayser et al. 2013). Changes in mood, behaviour or personality are also common early features in children and adolescents. Symptoms can include anxiety, agitation, bizarre behaviour, catatonia, delusional or paranoid thoughts and visual or auditory hallucinations, accompanied by memory loss (Irani et al. 2010a). It has been recognised that a minority of individuals only present with one or few symptoms, usually psychosis or seizures (Kayser et al. 2013; Niehusmann et al. 2009). This raises the possibility of a partial or attenuated syndrome, with a predominance of psychotic symptoms, accompanied by few or no other clinical characteristics of NMDAR antibody encephalitis.

An observational study by Kayser et al. found 4 % of cases of NMDAR antibody encephalitis presented with *isolated* psychiatric symptoms. This rose to 28 % of a group experiencing relapses. Seventy-four percent had delusional thoughts, 43 % had auditory or visual hallucinations and 57 % had aggressive behaviour. Seventy percent had a mood component to their presentation including mania, mood lability, impulsivity, disinhibition and depressive or nonspecific mood changes (Kayser et al. 2013). The clinical picture has broadened further with a recent study finding 2 % of cases of post-partum psychosis had NMDAR antibodies (Bergink et al. 2015).

### Effects of immunotherapy

Most patients with a diagnosis of NMDAR antibody encephalitis experience a substantial improvement in their symptoms when treated with immunotherapy (first line: steroids, intravenous immunoglobulin (IVIg), plasma exchange; second line: rituximab, cyclophosphamide) and/or tumour removal (Titulaer et al. 2013), with early treatment a predictor of a good outcome (Kayser et al. 2013). Nevertheless, a majority of patients experience persistent subjective cognitive deficits or have deficits on cognitive testing including memory impairment and executive dysfunction (Finke et al. 2012). Memory deficits correlate with hippocampal damage on MR imaging, the extent of which is predicted by disease severity and duration, highlighting the importance of early diagnosis and appropriate treatment (Finke et al. 2012, 2015).

### Potential mechanisms underlying the psychiatric effects of NMDAR N1 antibodies

**Fig. 1:**
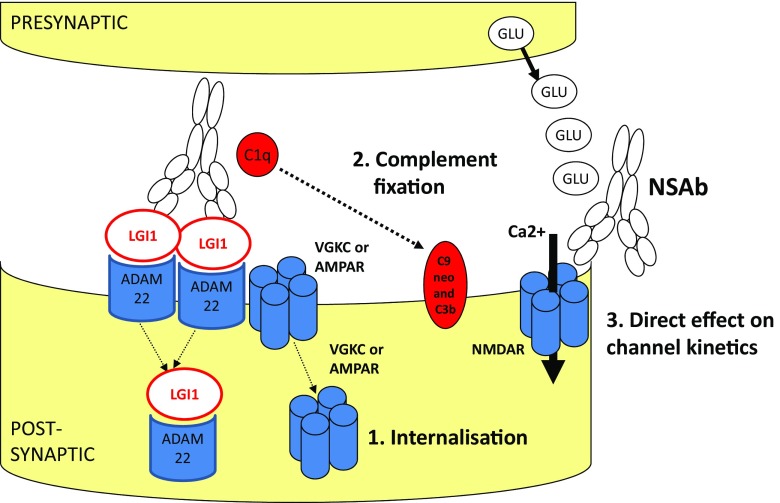
Potential mechanisms of action of NSAbs. Specific NSAbs represented here are examples only and multiple mechanisms may be shared by different NSAbs. Note that NSAbs are likely to have relevant downstream effects on intraneuronal signalling, compensatory changes in the expression of other surface proteins and effects on larger-scale neuronal network function. Figure reproduced with kind permission from Varley et al. (Varley et al. 2015)

Of all NSAbs, the mechanisms underlying the effects of NMDAR NR1 antibodies have been most extensively investigated and mimic many of the mechanisms established originally in the study of pathogenic antibodies directed against the acetylcholine receptor in myasthenia gravis, the prototypical antibody-mediated neurological disorder. Although the epitope is located within the N-terminus that also contains the glycine binding site, it has been demonstrated that NMDAR antibodies do not have a direct action at the receptor (Moscato et al. 2014). Rather they cause a reversible, time- and dose-dependent internalisation of cell membrane-bound NMDARs with a subsequent decrease in synaptic and extrasynaptic receptor density and a reduction in NMDAR-mediated currents and synaptic plasticity (Fig. 1 and Table 2).

Glutamate dysfunction, in particular NMDAR hypofunction, is thought to be central to the pathophysiology of schizophrenia (Howes et al. 2015). NMDAR antagonists such as phencyclidine (PCP) and ketamine are able to induce psychotic and cognitive symptoms resembling those seen in psychotic disorders (Javitt 2007) and are similar to those described in NMDAR antibody encephalitis. Furthermore, PCP can stimulate agitation and dissociative states including reduced responsiveness with catatonic features (Javitt and Zukin 1991), well-described in NMDAR antibody encephalitis. NMDAR antibodies also cause an increase in extracellular glutamate (Manto et al. 2010), an effect directly comparable to that of the non-competitive NMDAR antagonist ketamine (Liu and Moghaddam 1995). The psychotic symptoms associated with ketamine use are directly linked to cortical glutamate levels (Stone et al. 2012), suggesting that this could also be a mechanism by which NMDAR antibodies cause psychosis.

Partial NR1 knockout mice display schizophrenia-related behaviours, including cognitive impairment (Belforte et al. 2010). Multiple genes associated with schizophrenia are related to the NMDAR and associated synaptic proteins (Hall et al. 2015; Kirov et al. 2012; Timms et al. 2013). Post-mortem data provide evidence for abnormalities in the NMDAR in patients with schizophrenia (Rubio et al. 2012). There is also in vivo evidence for reduced NMDAR in the hippocampus of unmedicated patients with schizophrenia (Rubio et al. 2012; Pilowsky et al. 2006). Furthermore, there is some limited evidence that drugs modulating the NMDAR are effective in schizophrenia (Stone 2011).

Planaguma and colleagues developed an animal model of NMDAR encephalitis in which patient CSF IgG was administered to mice via intraventricular infusion over 14 days. The mice had memory impairments, anhedonia and depressive behaviour, but there were no effects on anxiety or locomotor activity (Planaguma et al. 2015). From the neuropsychiatric perspective, while this model shows clear in vivo effects of NMDAR antibodies, it does not provide a good fit with the phenomenology of NMDAR antibody encephalitis, a condition frequently characterised by agitation, anxiety and affective lability or even mood elevation. Further, despite psychosis being the most common psychiatric feature, the authors did not assess effects on paradigms used in animal models of psychosis. Given the absence of any clear epileptic seizures, autonomic dysfunction or movement disorders in the mice, it is also unclear how valid a model this is of NMDAR antibody encephalitis more broadly.

### Other NMDAR antibodies

NMDAR NR2 antibodies, as detected using ELISA, have been implicated in neuropsychiatric systemic lupus erythematosus (SLE) (Lauvsnes and Omdal 2012), where they have also been associated with a reduction in hippocampal volume (Lauvsnes et al. 2014). No published work to date has addressed whether there is overlap between the antibodies in this population and with the NR1 antibodies implicated in NMDAR encephalitis, but the detection methods are sufficiently different to suggest no overlap exists.

More recently, neuropsychiatric presentations have also been associated with IgA and IgM NMDAR antibodies. NMDAR IgA and IgM antibodies have been found in patients with progressive cognitive dysfunction and dementia (Pruss et al. 2012a; Doss et al. 2014), and IgM antibodies have been identified in cases of bipolar affective disorder and psychosis (Choe et al. 2013; Hammer et al. 2014). These studies also provide growing evidence for their pathogenicity in vitro, but this is not as robust as for the IgG subtype. However, their presence in the CSF of some patients may suggest that they have pathogenic potential (Doss et al. 2014).

## Voltage-gated potassium channel (VGKC) complex antibodies

Initially described in association with peripheral nerve hyperexcitability, antibodies that immunoprecipitated the alpha-dentrotoxin-sensitive VGKC alpha subunits were soon (Liguori et al. 2001) recognised in Morvan’s syndrome, a condition featuring both peripheral nerve and neuropsychiatric symptoms including anxiety, obsessional behaviour, sleep-wake disruption and psychosis (Irani et al. 2012). Limbic encephalopathy in association with VGKC complex antibodies was first described in 2001 (Buckley et al. 2001). Memory deficits, disorientation and medial temporal lobe seizures predominate, although psychiatric symptoms are often present and may occasionally be the presenting feature (Thieben et al. 2004). Psychiatric symptoms include, in order of decreasing frequency, personality change, depression, anxiety, visual hallucinations, spells and delusions (Somers et al. 2011). Sleep abnormalities are common (Cornelius et al. 2011).

Some patients with VGKC complex antibody-associated encephalopathies present initially to psychiatric services, although this proportion is smaller than in NMDAR encephalitis. Patients with VGKC complex antibodies tend to be older than those with NMDAR antibodies (Paterson et al. 2014). In addition to the prominence of cognitive and psychiatric symptoms, a number of cases have now been described in which VGKC complex antibodies have been associated with a predominantly psychiatric phenotype, usually comprising a schizophreniform or more polymorphic psychosis with varying degrees of cognitive deficit (Somers et al. 2011; Zandi et al. 2011; Parthasarathi et al. 2006; Tang et al. 2015), although it is unclear whether this includes cases in which the antibodies are causally relevant (see below) (Paterson et al. 2014).

### Antibodies to VGKC-‘complexed’ proteins

VGKC complex antibodies were initially detected using a radioimmunoassay which detects immunoprecipitation of alpha-dendrotoxin-labelled VGKCs from mammalian brain tissue. Subsequent inductive biochemical experiments have demonstrated that some VGKC antibodies are not usually directed against the VGKCs themselves but against one or more of three proteins strongly complexed with the VGKC in mammalian brain: LGI1, CASPR2 and contactin-2 (Irani et al. 2010b).

LGI1 antibodies are more frequently associated with epilepsy and encephalopathy syndromes, while CASPR2 antibodies are more commonly associated with disorders of peripheral nerve hyperexcitability including Morvan’s syndrome. This is consistent with the CNS-predominant expression of LGI1, whereas CASPR2 is expressed densely at juxtaparanodes in the CNS and PNS. A highly distinctive seizure semiology described as faciobrachial dystonic seizures (FBDS) precedes the development of frank encephalopathy in a proportion of cases with LGI1 antibodies (Irani et al. 2011). Both LGI1 and CASPR2 antibody-associated encephalopathies respond well to prompt immunotherapy, although residual amnestic deficits are common (Butler et al. 2014) and may occur as a function of time to treatment in LGI1 antibody encephalopathy (Irani et al. 2013).

Functionally, LGI1 antibodies specifically block the binding of LGI1 to ADAM22/ADAM23 with a corresponding decrease in synaptic AMPAR density (Ohkawa et al. 2013). However, given the clear phenotypic differences between LGI1 antibody encephalitis and AMPAR antibody encephalitis (see below), it is likely that LGI1 antibodies find clinical expression through mechanisms additional to a reduction of synaptic AMPAR numbers or function. In this regard, there is evidence from a single study that LGI1 antibodies may also act via direct interference with the VGKCs, potentiating hippocampal mossy fibre to CA3 pyramidal cell transmission (Lalic et al. 2011). This is also supported by the frequent detection of LGI1 antibodies using the VGKC complex radioimmunoassay.

Intriguingly, mutations in the LGI1 gene in humans are associated with lateral temporal lobe epilepsy syndromes with prominent auditory and sometimes visual and even psychic auras (Striano et al. 2011). The mutations are not however associated with increased rates of primary psychiatric illness. Mutations in the gene encoding CASPR2, CNTNAP2, are associated with schizophrenia, epilepsy and autism (Alarcon et al. 2008; Friedman et al. 2008).

## AMPA receptor antibodies

Antibodies to the GluR1 and GluR2 AMPAR subunits have been associated with limbic encephalitis characterised by short-term memory deficits, emotional and behavioural changes and seizures. Initially described in ten patients from a series of 109 cases of limbic encephalitis, the majority of cases were older women and seven had tumours (of the lung, breast or thymus). Nine patients responded to immunotherapy or oncological therapy. However, neurological relapses without tumour recurrence were frequent (Lai et al. 2009).

More recently, two studies retrospectively tested for AMPAR antibodies in samples from patients with more varied clinical presentations (Hoftberger et al. 2015; Dogan Onugoren et al. 2014). One included 4,819 samples from patients with a wide range of neuropsychiatric presentations. Three were positive for GluR2 AMPAR antibodies, with one having an ovarian tumour. All presented with memory deficits, and two had psychiatric symptoms, including anxiety and mood symptoms (Dogan Onugoren et al. 2014). The other study contained 10,573 patients presenting with suspected encephalitis or paraneoplastic syndromes and found 22 patients positive for AMPAR antibodies. Six had psychotic symptoms as part of their presentation. One of these patients presented with a week of an isolated psychotic illness, before developing neuroleptic malignant syndrome in response to antipsychotic medication. Most patients had a tumour and a good or partial response to immunotherapy or surgery (Hoftberger et al. 2015).

Case studies further highlight the potential for presentations with almost exclusively psychiatric symptoms. Two describe patients presenting with acute behavioural changes (agitation, confusion and aggression). One had an associated dysphasia, and the other had a past history of thymoma. Both showed a poor response to neuroleptics but a good response to corticosteroids (Graus et al. 2010). The third describes an older woman presenting with headache, confusion, hallucinations and paranoia. Her symptoms responded to IVIg therapy but relapsed (Elamin et al. 2015). These case studies raise the possibility of a predominantly psychiatric presentation with limited neurological associations, potentially amenable to immunomodulation (Graus et al. 2010). Other case studies describe psychiatric symptoms but as part of a more varied clinical presentation (Bataller et al. 2010; Wei et al. 2013).

### Potential mechanisms of AMPAR antibodies

AMPAR antibodies reversibly reduce the total surface amount and synaptic localization of GLuR1 and GLuR2 containing AMPARs by internalisation and degradation of AMPAR clusters (Lai et al. 2009; Peng et al. 2015), with an accompanying reduction in AMPAR-medicated currents (Gleichman et al. 2014; Peng et al. 2015). Together, these changes appear to result in a compensatory decrease of inhibitory synaptic transmission and increase in intrinsic excitability of neurons.

AMPARs are ionotropic glutamate receptors involved in both fast glutamatergic neurotransmission and activation of NMDARs (Malinow and Malenka 2002). By the nature of their interaction with NMDARs, it is possible that changes in the activity of these receptors could induce psychotic symptoms. They are important in synaptic plasticity, learning and memory (Malinow and Malenka 2002; Kessels and Malinow 2009; Keifer and Zheng 2010). Post-mortem studies have found alterations in the expression and binding sites of AMPAR in schizophrenia (Rubio et al. 2012; Meador-Woodruff and Healy 2000) and mood disorders (Freudenberg et al. 2015; Alt et al. 2006; Gibbons et al. 2012). Mice lacking the AMPA GLuR1 receptor display schizophrenia-related behaviours (Wiedholz et al. 2008) and depressive-related behaviours (Chourbaji et al. 2008).

AMPAR potentiating drugs have moderate effect on cognitive symptoms in schizophrenia when co-administered with antipsychotics, but this is the subject of an ongoing research (Goff et al. 2001; Menniti et al. 2013). There is also evidence for an antidepressant effect in animal studies, and clinical trials are in progress (Freudenberg et al. 2015; Alt et al. 2006; Li et al. 2001).

## GABA receptor antibodies

Antibodies to the GABA_B_ receptor have been described in limbic encephalitis with seizures and associate with an underlying lung or neuroendocrine tumour in approximately half of patients (Hoftberger et al. 2013). The psychiatric features described as part of the limbic encephalitis (memory loss, confusion, personality change; psychosis occurring in about a third (Lancaster et al. 2010)) do not differentiate GABA_B_R encephalitis from other NSAb-associated limbic encephalitides.

GABA_A_R antibodies targeting isoforms containing α_1_ or β_3_ subunits were described in a series of 18 patients, of whom six had high titre antibodies which were also detectable in CSF and presented with severe encephalitis and refractory seizures. The other subjects, with lower titre antibodies detectable in serum only, had a more varied clinical presentation and often had other serum NSAbs (Petit-Pedrol et al. 2014). Pettingill and colleagues retrospectively identified GABA_A_R antibodies specific for the α_1_ and γ_2_ subunits in the sera of 40 of 2,046 patients with varied clinical features whose sera were negative for other NSAbs (Pettingill et al. 2015). Surprisingly, around half were IgM antibodies but all bound to live neurons. This approach has resulted in more varied clinical associations: psychiatric features were present in 5/15 for whom clinical data were available—these included patients with established psychiatric diagnoses of schizophrenia, obsessive-compulsive disorder and ‘catatonia of unknown origin’. This last patient was a 17-year-old male who presented with anxiety, obsessionality and psychosis symptoms before developing catatonic motor symptoms which improved with plasma exchange on two separate occasions.

### Potential mechanisms of GABAR antibody effects

GABAR hypofunction is a plausible mechanism for the generation of psychiatric symptoms in these patients. Polymorphisms in genes encoding GABARs have been associated with multiple psychiatric presentations, including autism, anxiety disorders and psychotic disorders. Reduced GABA_A_R availability is associated with psychotic disorders (Frankle et al. 2015) and clinical risk for such disorders (Kang et al. 2014).

## Dopamine receptor antibodies

Reactivity to D2R was described using ELISA and western blot with antibodies purified from individuals with basal ganglia disorders associated with streptococcal infection, including Sydenham’s chorea and paediatric autoimmune neuropsychiatric disorders associated with streptococcal infections (PANDAS), and as such the psychiatric associations encompassed anxiety, obsessions, compulsions and tics (Brimberg et al. 2012). In contrast to most of the NSAbs described here, these antibodies appeared to potentiate rather than antagonise their target receptor-based signalling (Cox et al. 2013; Brimberg et al. 2012). This is in keeping with the considerable evidence for striatal hyperdopaminergia in tic disorders and obsessive-compulsive disorder (OCD) (Denys et al. 2013). Increased levels of dopamine D1 receptor antibodies have since been reported in OCD and Tourette syndrome (Cox et al. 2015), but as these were detected with methods that offer less natively conformational epitopes than CBAs their pathogenicity is unclear.

Studies using CBAs confirmed reactivity with *cell surface* D2Rs (providing stronger evidence of pathogenicity) in Sydenham’s chorea but not in PANDAS. These NSAbs were also found in Tourette syndrome and paediatric basal ganglia encephalitis with prominent psychiatric symptoms (also termed paediatric dyskinetic encephalitis lethargica) and first-onset paediatric psychosis (Pathmanandavel et al. 2015; Dale et al. 2012), expanding the psychiatric phenotype to include psychosis. Whether these D2R antibodies stimulate the D2R has not yet been demonstrated, but this would be consistent with the now considerable evidence for dopaminergic overactivity in psychosis (Howes et al. 2012). A large study of adults with schizophrenia experiencing an acute psychotic episode however failed to find D2R antibody positivity in this group (Muller et al. 2014), although notably the CBA methodology differed considerably from the previous study.

## Other antigen targets

Glycine receptor and voltage-gated calcium channel (VGCC) antibodies have been consistently associated with characteristic neurological symptoms but are rarely associated with psychiatric symptoms. This is somewhat surprising in the case of VGCC antibodies given the compelling evidence for the role of calcium ion channel gene defects in multiple psychiatric disorders (Cross-Disorder Group of the Psychiatric Genomics, C 2013). The neuroanatomical specificities of the NSAbs or their cognate antigens may be relevant here, with glycine receptor antibodies predominantly affecting brainstem structures and the described CNS specificities of VGCC antibodies to date appearing to favour the cerebellum (Burk et al. 2010; Fukuda et al. 2003).

To date, fewer than 30 patients have been described with an encephalopathy syndrome associated antibodies to DPPX, a regulatory subunit of A-type (rapidly inactivating) potassium channels (Boronat et al. 2013; Tobin et al. 2014). Although initially described in connection with prominent gastrointestinal symptoms, presumably due to the high levels of DPPX in the myenteric plexus, the disorder can have CNS-only manifestations. From a neuropsychiatric viewpoint, in the largest series described to date, 80 % had amnesia, 40 % delirium and 20 % had each of psychosis and depression (Tobin et al. 2014).

Four patients have been described with limbic encephalitis and antibodies to mGluR5. Psychiatric symptoms including psychosis and affective and personality changes were prominent; three had Hodgkin lymphoma (Lancaster et al. 2011; Pruss et al. 2014; Mat et al. 2013). The effect of the NSAbs on mGluR5, which is thought to regulate NMDAR-dependent signalling, is unknown. Notably, dysfunction of mGluR5 is implicated in the pathogenesis of glutamate dysfunction in psychiatric disorders including schizophrenia (Matosin et al. 2015). Compounds that act on the mGLuR are being developed for the treatment of schizophrenia (Patil et al. 2007), but it is currently unclear if they are effective.

## Non-receptor-based effects

While this review has emphasised the theoretical utility of viewing NSAb function from a pharmacological perspective, it is important to note that NSAbs have other effects consistent with their role as immune effector molecules. In LGI1 antibody-mediated encephalitis but not in NMDAR antibody encephalitis, for example, there is consistent evidence of complement deposition and associated cell death in patient brain tissue (Bien et al. 2012).

Pathological studies of NMDAR antibody encephalitis have demonstrated the presence of activated microglia (Dalmau et al. 2008). Consistent with this, microglial activation in a man with NMDAR antibody encephalitis has been demonstrated in vivo using a TSPO-specific PET ligand; the degree of microglial activation was found to correlate with clinical severity and antibody titre (Jensen et al. 2015).

Surprisingly, little work has focussed on the role of inflammatory mediators such as cytokines and chemokines in NSAb-associated CNS disease. One study found evidence of involvement of the Th-17 pathway (Ulusoy et al. 2012), but this requires replication and further work to elucidate potential differences in the inflammatory milieu associated with different NSAbs. Given the increasing recognition of the role of inflammation in primary psychiatric disease, studies of this kind may offer further insights of the pathogenesis of antibody-mediated psychiatric symptoms across diagnoses.

The importance of the inflammatory effects of NSAbs in human disease is unclear and is likely to vary according to the antigen target, immunoglobulin subtype (e.g. IgG4 antibodies are thought to act mainly through effects on receptor function (Huijbers et al. 2015)) and disease stage (for example, inflammatory effects may contribute to the residual symptomatology seen in many post-encephalitic patients, as is likely the case in hippocampal atrophy following LGI1-antibody encephalopathy (Malter et al. 2014).

## Evidence for NSAbs in primary psychiatric disease

This review has detailed how NSAbs can cause psychiatric symptoms as part of a wider constellation of neurological symptoms in autoimmune encephalopathies. There is also now an understandable vogue to similarly study the neuropsychiatric features of neurological disorders such as epilepsy or dementia, in which NSAbs have been variably linked with the presence of psychiatric symptoms, in particular psychosis (Doss et al. 2014; Busse et al. 2014; Ekizoglu et al. 2014). One of the most controversial areas of psychiatric research today concerns the *further* question of whether NSAbs have a causal role in psychiatric disorders such as schizophrenia, affective disorders or autistic spectrum disorders.

### Controversies and limitations of current evidence

Despite a considerable research effort, there has been relatively disappointing progress in the identification of novel biological treatments for psychiatric disorders. The possibility of NSAb-mediated psychiatric disease has been met with great enthusiasm probably because it raises the prospect of an entirely new class of therapy for at least a subset of these highly disabling illnesses.

There are presently a number of case studies and series, albeit uncontrolled, demonstrating immunotherapy-responsive psychiatric presentations associated with NSAbs (Table 1). The main controversies remaining are whether serum antibodies alone are sufficient to diagnose an antibody-mediated CNS disease and whether these cases are a rarity or whether they might account for a significant proportion of individuals with a given psychiatric diagnosis. Evidence for either possibility is inconsistent and the literature has been beset with debate around the most appropriate methodologies for establishing the presence of *causally relevant* antibodies.

Most research has focussed on schizophrenia and psychosis, probably because of the frequency with which psychosis features in autoimmune encephalopathies but also because of (a) the theoretically appealing links between neurotransmitter receptors as NSAb targets and the suggested role of these neurotransmitters (e.g. NMDAR, AMPAR, D2R) in the pathogenesis of psychotic disorders, (b) epidemiological evidence highlighting a strikingly increased risk of psychosis in people with autoimmune disorders and vice versa (Benros et al. 2011, 2014) and (c) powerful genome-wide evidence of the centrality of immune-related genes in psychosis (Schizophrenia Working Group of the Psychiatric Genomics, C 2014).

Taking NMDAR antibodies in psychotic disorders as the most studied example, some studies using live, non-permeabilised CBAs have demonstrated increased rates of NMDAR antibodies in psychotic disorders (Pathmanandavel et al. 2015; Zandi et al. 2011). Studies employing fixed and permeabilised CBAs however have broadly found similar prevalences of NMDAR antibodies in patients and in controls (Dahm et al. 2014; Hammer et al. 2014), while studies using multiple assays have failed to find any NMDAR antibodies in psychotic patients (Masdeu et al. 2012). Notably, as well as the inter-assay variability, patients in each of these studies differed markedly in terms of chronicity and acuity of illness (see (Pollak et al. 2014)).

There are a number of areas of controversy regarding testing for NSAbs, particularly in populations with ‘atypical’ presentations—that is, whose symptoms differ from the canonical encephalopathy syndromes described above. (1) The necessity of testing CSF as opposed to serum alone is disputed. Intrathecal antibody synthesis appears to be common for some NSAbs but not others (e.g. common in NMDAR antibody encephalitis but not LGI1 antibody encephalitis (Malter et al. 2013))—but whether antibodies are detectable in CSF may depend on disease stage and the specificities of the assay used such as the dilutions at which serum and CSF are tested (see Irani et al. for further detail (Irani et al. 2014)). (2) The decision to use fixed as opposed to live CBAs may impact the outcome of prevalence studies since fixation of cells modifies the antigen and permeabilises the cell membrane. The latter may expose intracellular antigens and therefore potentially detect causally irrelevant antibodies. (3) The association between psychiatric disorders and NSAbs may depend on the seropositivity threshold employed in the CBA, with one meta-analysis indicating higher odds of NMDAR antibody seropositivity in psychotic and affective disorders at lower titres (Pearlman and Najjar 2014). Most CBAs ascertain titre by performing serial dilutions and ascertaining the lowest dilution at which immunofluorescence is detected. More finely grained quantitative CBA results are possible using fluorescence-activated cell sorting (Amatoury et al. 2013), but so far, this is limited to a small number of laboratories and the sensitivity of the method has in one study been shown to be inferior to microscopy (Ramberger et al. 2015). (4) Since NMDAR antibody encephalitis is associated with IgG antibodies, it had been assumed that only these antibodies had functional effects and hence could cause disease. However, as mentioned in the section on NMDAR antibodies, there is now increasing evidence that antibodies of IgA and IgM subclasses may have a pathogenic role in psychiatric and neurodegenerative disorders (Pruss et al. 2012a; Doss et al. 2014; Choe et al. 2013; Hammer et al. 2014). (5) Finally, the necessity of confirming a CBA result with other techniques to demonstrate immunoreactivity, such as immunohistochemistry or assessment of antibody binding to cultured neurons, is also debated (Zandi et al. 2015; Kayser 2015).

### Future directions

It is clear at this stage that studies purporting to show an increased prevalence of a given NSAb in a particular psychiatric patient group cannot in isolation establish that these antibodies have any pathogenic role. Although the recognised frequencies of these antibodies in a variety of disease and healthy populations (Dahm et al. 2014) suggest that thorough epidemiology should be revisited with detailed clinical correlations. One potentially powerful argument for the causal relevance of NSAbs in individuals with a psychiatric diagnosis comes from case reports and series demonstrating psychiatric improvement following immunotherapy in patients with NSAbs and a primary psychiatric diagnosis (Zandi et al. 2011, 2014)—although this could relate to an antibody-independent immunological process for which the antibody is a non-causative biomarker. Randomised controlled trials of immunotherapy in these patient groups are necessary to establish efficacy before standard psychiatric treatment is substantially changed, however.

Another argument for pathogenicity of NSAbs in psychiatric groups comes from increasing evidence of in vivo and in vitro functional effects of antibodies from patients with a primary psychiatric disorder (Choe et al. 2013; Hammer et al. 2014). That is, if the effects on receptor number, function and cell signalling, or indeed animal behaviour, resemble those seen with antibodies from patients with encephalitis (Table 2), then the case for pathogenicity of these NSAbs would appear at least equal to that in cases of encephalitis. However, if the antibodies are derived from the serum, it may be that the CNS is never accessed by these IgG species.

An alternative question concerns whether, when found in individuals with psychiatric disorders, NSAbs are a secondary phenomenon, emerging as part of an immune response to whichever pathogenetic process is driving the disease process. Importantly, this does not exclude the possibility that NSAbs, even if secondary, can influence clinical phenotype and/or disease course. One way to distinguish this possibility from a more straightforward causal role would be if NSAbs were not detected in serum samples from individuals at high risk for psychiatric illness (e.g. the ‘at-risk mental state’ for psychotic disorders) but became detectable after the onset of clinical disease. Alternatively, NSAbs may have prognostic significance in psychiatric disorders, akin to the prognostic role of other autoantibodies in many disorders throughout medicine.

Much work remains to be done to elucidate the conditions required for NSAbs to be produced and to cause CNS dysfunction, with potential relevance for psychiatry. Some authors have highlighted the importance of blood–brain barrier (BBB) dysfunction in determining whether peripheral antibodies can reach the CNS and exert pathogenic functional effects (Hammer et al. 2014; Huerta et al. 2006; Levin et al. 2010). With increasing recognition that the majority of NSAb seropositivity is not associated primarily with malignancy, attention has also turned to infection as a potential antecedent of pathogenic NSAb formation; possible mechanisms include nonspecific adaptive immune response to neuronal damage and molecular mimicry (Bogdanos et al. 2013). Particular links between the formation of glutamate receptor antibodies and influenza (Hammer et al. 2014), HSV-1 (Pruss et al. 2012b; Armangue et al. 2014), EBV (Xu et al. 2011) and other viral (Koustova et al. 2001) infections have been demonstrated. Interestingly, serological evidence of viral or other infections has also been linked with risk for psychosis (Wang et al. 2011; Amminger et al. 2007; Yolken and Torrey 2008) or with particular phenotypes within psychosis populations including cognitive impairment (Shirts et al. 2008) and neuroimaging abnormalities (Schretlen et al. 2010; Prasad et al. 2011); whether NSAbs might mediate this relationship in a subset of cases has not been explored.

## Therapeutic considerations

NSAbs are immune effector molecules which target specific CNS receptor targets or other antigenic targets which directly impact upon receptor function. Therapeutically, two broad approaches can therefore be considered: immunological and receptor-based therapies. Precedent for this can be found in the treatment of myasthenia gravis, in which most patients have antibodies to cell surface nicotinic acetylcholine receptors. Therapy for the disease is usually a combination of immunotherapy targeting the antibodies and cholinergic therapy aimed at restoring the cholinergic balance at the neuromuscular junction. Similarly, targeted therapies for NSAb-mediated disease may similarly be employed simultaneously on two fronts.

Current immunotherapy for NSAb-mediated CNS disease can be divided into first-line and second-line therapies. First-line therapies include oral or intravenous steroids for nonspecific immunosuppression, plasma exchange and intravenous immunoglobulins. Second-line therapies include cyclophosphamide, mycophenolate mofetil and B cell-specific depletion including the anti-CD20 monoclonal antibody rituximab. Other immunological therapies currently in use in rheumatology and neurology clinics may yet have application in NSAb-mediated disease. Promising compounds are likely to be those specifically targeting B cells and plasma cells and associated antibody production, either directly or indirectly (e.g. tocilizumab, which targets the IL-6 receptor on B cells (Irani and Vincent 2015)). Immunotherapies are associated with a number of potentially serious side effects and should be used with caution in the patients most likely to benefit. Side effects include increased risk of infection (including serious opportunistic infections of the CNS and systemically) with immunosuppressive treatments, fever, headache and anaphylaxis with intravenous immunoglobulins and cardiovascular instability with plasma exchange. Interestingly, steroid-induced psychosis is very rarely reported in the treatment of autoimmune encephalopathies. This may be because psychosis is often a presenting symptom of the illness itself, and hence exacerbations are more likely to go unnoticed or ascribed to illness progression or potentially may be related to differential mechanisms underlying behavioural disturbance in these individuals.

One example of the second, receptor-based approach has been described in a therapeutic open-label case study by Heresco-Levy and colleagues who gave d-serine to a female with an NMDAR antibody-positive woman with a diagnosis of chronic treatment-refractory schizophrenia. She also had cortical and subcortical white matter MRI hyperintensities and runs of ‘extreme delta brush’ on EEG (a pattern thought to be pathognomonic of NMDAR antibody-mediated CNS disease). Following treatment with d-serine, which is a NMDAR co-agonist thought to enhance NMDAR function by increasing the frequency of channel opening, the patient’s EEG normalised and her psychosis improved (Heresco-Levy et al. 2015).

Another future potential receptor-based approach, arising from the work of Diamond and colleagues on NMDA NR2 antibodies in neuropsychiatric SLE, involves the synthesis and systemic administration of a d-peptide which prevents binding of antibody to its binding site without directly affecting receptor function: in mice, these were well tolerated and blocked the neurotoxic effects of NR2 NMDAR antibodies (Huerta et al. 2006). Approaches using conformational epitopes would be required in autoimmune encephalopathies, as the antibodies recognise non-linear native confirmations of the target proteins. Other strategies aimed at blocking antibody-antigen interaction are in development in neuromyelitis optica and may also have application in NSAb-mediated disorders (Papadopoulos et al. 2014).

## Antibodies as therapeutic agents

Speculatively, and bearing in mind the growth of antibody-based therapeutics in medicine generally, one could foresee how recent work on NSAbs could facilitate the development of functionally active antibodies to specific receptor targets as an emergent therapeutic strategy within neuropsychopharmacology. There is limited evidence that in some circumstances NSAbs could have a protective function: Zerche and colleagues have demonstrated that pre-existent NMDAR antibodies limit lesion size following stroke in individuals with an intact BBB (Zerche et al. 2015). Although the mechanism is not known, it may be that NMDAR-Ab-mediated receptor hypofunction limited glutamate-mediated excitotoxicity in these individuals.

## Conclusion

Increasingly, NSAbs are being reported in diverse clinical populations with a range of psychiatric phenotypes. We have presented convergent evidence that, outside of narrowly defined psychiatric populations at the very least, NSAbs are associated with a number of psychiatric syndromes, including psychosis, tic and mood disorders, and evidence that immunotherapy to treat the NSAbs is effective in treating the psychiatric symptoms. However, several areas of controversy remain, in particular whether the expanding numbers of studies reporting increased prevalence of NSAbs in primary psychiatric populations might require us to reconceptualize the nature of a subset of these disorders. Studies demonstrating whether NSAbs from psychiatric patients have functional effects in vivo and in vitro are urgently required to address this question, as are well-controlled trials of immunological therapies in appropriately selected antibody-positive patients.

NSAbs are potent effector molecules within the CNS that can have profound effects on neuronal receptor function. Their high affinity and remarkable specificities could offer opportunities to study the biology of CNS-active molecules in humans. A combined psychopharmacological and immunological approach to understanding their mechanisms of action is necessary to understand both the neurobiological basis of psychiatric symptoms as well as to develop new treatment strategies that will arise from the inevitably improved understanding of antibody-antigen interaction within the CNS.
